# The role of expansion and adaptability of face-space for individual differences in face identity processing

**DOI:** 10.1098/rsos.240879

**Published:** 2025-01-22

**Authors:** Linda Ficco, Meike Ramon, Anna Schroeger, Jürgen M. Kaufmann, Stefan R. Schweinberger

**Affiliations:** ^1^ Department of General Psychology and Cognitive Neuroscience, Friedrich Schiller University, Jena, Germany; ^2^ Faculty of Social and Political Sciences, Institute of Psychology, University of Lausanne, Lausanne, Switzerland; ^3^ International Max-Planck Research School for the Science of Human History, Jena, Germany; ^4^ Department of Biological Psychology and Cognitive Neurosciences, Friedrich Schiller University, Jena, Germany; ^5^ Department of Experimental Psychology, Justus Liebig University Giessen, Giessen, Germany; ^6^ Department for the Psychology of Human Movement and Sport, Friedrich Schiller University, Jena, Germany; ^7^ German Center for Mental Health (DZPG), Site Jena-Magdeburg-Halle, Jena, Germany

**Keywords:** typicality, face-space, norm-based coding, face detection, face recognition, individual differences

## Abstract

Individuals can strongly vary in their ability to process face identity. Understanding the mechanisms driving these differences is important for theoretical development, and in clinical and applied contexts. Here we investigate the role of face-space properties in relation to individual face identity processing skills. We consider two fundamental properties of face-space: *expansion* (how distant from each other similar faces are located in such space) and *adaptability* (the degree to which these distances change over time). Fifty-two participants performed a face detection task, with faces systematically varying in their location in face-space, and a comprehensive face identity processing test battery. We replicate previous results indicating a detection advantage for typical, as compared with distinctive faces. Critically, we find that neither our measure of face-space expansion nor that of face-space adaptability are related to individual face processing abilities. While future studies might benefit from the use of more sensitive measures of face-space properties, these results suggest that the two examined here do not contribute to individual differences in face processing abilities as previous studies suggest.

## 1. Introduction

### 1.1. Factors explaining interindividual differences in face processing

Faces are highly relevant for social interaction. We can remember thousands of faces [1], and are likely exposed to hundreds of thousands throughout our life, depending on social factors (network size, mobility, personality traits, profession, etc.). Standardized tests of face identity processing ability (measuring perception, or perception and memory) reveal remarkable variability, from individuals who exhibit face blindness (prosopagnosia; see [2,3]) to Super-Recognizers [4–8], with the majority falling somewhere in between. Which mechanisms underlie such individual differences in face processing has become a question of fervent scientific interest over the last decade [9–12].

Two main streams of behavioural research have tried to answer this question. The first has investigated the role of *social-personal* variables, such as personality and autistic traits [13–20], or social network size [20] and gregariousness [16]. The second stream of research has focused on purely *visual* factors, such as visual acuity [21–23], contrast sensitivity [22,24–27], processing of spatial frequency information [28,29] or holistic processing [30–34]. Therefore, at least partly, individual differences in face processing seem to be driven by general visual abilities, as also indicated by studies reporting correlations between face and object recognition tests (see third section in [35]). However, these correlations are consistently smaller than those between face tests [35], suggesting individual differences in face processing are genuine, and possibly reflect a general ‘f factor’ in perception [36]. This motivated us to include only face processing tests in our battery. While determining the contribution of these two types of factors to face recognition is likely premature and beyond the scope of this article, it is interesting to note that a third, intermediate factor, related to how a face is *mentally represented* in relation to all previously seen faces, has so far received relatively little attention (with notable exceptions, such as [37–39]).

### 1.2. The role of mental face representations

Our perception of a given face depends on the faces we have been exposed to in the past. ‘Face-space’ models [40,41] recognize and integrate this influence. Specifically, they suggest that encountered facial identities are encoded in a representational space at a certain distance from other identities, depending on its overall similarity to them [40,42,43]. The dimensions of this space not only represent variations of individual features (e.g. varying eye colour) but also variations of the several features that tend to co-occur (e.g. face ‘harshness’ versus ‘softness’). Theories differ in terms of their proposed structure of this mental face-space. In the norm-based multidimensional face-space model proposed by Valentine [40], each dimension is assumed to be normally distributed, and the means of all dimensions lie in the centre of the space, which represents a *weighted average* of all faces. Importantly, this representational space is dynamic. Temporary systematic shifts in the perceived stimuli (for instance, shifts induced via perceptual adaptation) can cause transient changes in the organization of this space and the corresponding weighted average face [44].

Beyond our individual face diets (i.e. everyday exposure to faces), our neural systems are also highly idiosyncratic. Consequently, individuals’ face-spaces will be shaped differently with exposure to novel stimuli, both as a function of time and diagnostic dimensions, giving rise to differently composed face-spaces across individual observers. The idea that individuals differ in their space recalibration speed has been investigated using perceptual after-effects [45]. These refer to changes in the perception of a stimulus after exposure to one or more stimuli that present systematic deviations from an average stimulus along one dimension. For example, a face resulting from a 50% morph of two identities A and B is perceived more as identity B after a few seconds of exposure to identity A, as compared with when the same stimulus follows a period of exposure to an identity-neutral face (e.g. identity C). The size of this perceptual shift is thought to reflect mechanisms of neural adaptation and can be informative about the flexibility of face representations [46]. Intriguingly, these adaptation studies consistently report positive relationships between the size of facial after-effects and face recognition skills [47–50]. This suggests that high performers’ perceptual systems retune faster than those of low performers to whichever dimensions of the current face diet best allow them to discriminate between identities [9].

The latter idea, that individuals differ not only in the adaptability but also in what we defined ‘expansion’ of their individual face-spaces (i.e. how distant from each other faces are represented) has received little attention. If individual face-spaces are organized around an (individual’s unique) average face, as predicted by norm-based multidimensional face space models, it should be possible to measure its expansion indirectly. For instance, image manipulation techniques can be used to systematically manipulate face averageness (such as morphing each face with an average, see e.g. [51,52]), and test the effects of this manipulation on participants’ behavioural and neural responses to these faces. Faces that are closer to the average are perceived as more typical [53,54], and typical faces are detected [55–57] and categorized faster [58] than distinctive faces, which are distant from the average. All this corpus of evidence suggests a processing advantage for typical faces, which we aim at replicating. At the same time, the relationship between individual processing advantages for typical faces in detection tasks and face identity processing skills remains open.

### 1.3. Face-space adaptability and expansion as predictors of individual performance

In this study, we primarily aim at investigating face-space *expansion* and face-space *adaptability* in relation to face identity processing skills. With our detection task we look to tap into the visual system’s preparedness to faces, in the sense that faster detection times in general would imply that someone’s brain is more versed to receive facial information. Consequently, larger differences in detection times between typical and distinctive faces in an individual as compared with others would indicate that typical faces are processed as even more ‘face-like’ and distinctive faces as even less ‘face-like’ in that individual. In other words, that this individual’s facial representations are more fine-grained, in that similar faces are more dissimilar from each other (face-space expansion). Finally, more marked shifts in these detection differences over time in an individual would indicate that what is ‘face-like’ for their brain has changed more rapidly for that person, as compared with others (face-space adaptability). The underlying assumption is that of a positive relationship between preparedness to facial information, especially fine-granularity and adaptability of facial representations and performance in face processing tests.

Recently Schroeger *et al*. [22] reported a positive relationship between face identity processing performers and effects of distance from average. Specifically, high performers, compared with low performers, show larger stepwise differences in P200 amplitudes in response to faces displaying systematic decreases in their distance from a face average. Therefore, we investigate whether the positive relation between face-space expansion and face processing performance extends to the behavioural level. Thus, we test, to our knowledge for the first time, whether the detection advantage for typical faces (shorter reaction times—RTs) reported in previous studies is related to face identity processing skills. If, as suggested by Schroeger *et al*. [22], high performers have a more fine-grained perception of changes in typicality (possibly because of larger distances between faces in their face-space), this should result in a positive relationship between the size of typicality detection advantage and test performance.

Moreover, building on past evidence of increased adaptability of an individual’s face-space over the course of an experiment in high face processing performers [48–50,59], we also test the extent to which differences in detection efficiency to distinctive and typical faces are predicted by individual face processing skills. Note that for this purpose we do not only measure RTs, but additionally calculate learning rates. This measure might capture the improvements of perception and detection of a face more meaningfully than RTs, as they represent detection efficiency changes throughout the task. First, in the context of face detection tasks, we expect larger learning rates (more efficient learning) for distinctive faces on average, based on the idea that these are subsequently better remembered [40,51,57,58,60–63]. Second, we expect that overall RT differences between typical and distinctive faces will decrease over time. Third and importantly, we expect *larger* differences in learning rates between typical and distinctive faces in high performers: although their face-space is expanded, so initially distinctive faces are located even further away from the centre of the space, we should see that by the end of the task both faces are detected with equal efficiency—indicating good recalibration of their face-space. Note that, from a methodological perspective, we consider that face identity processing performance can be highly task-dependent [64]. Accordingly, we quantify face identity processing abilities through multiple tests and use a composite [5,65], while also reporting exploratory analyses considering individual tests separately.

## 2. Material and methods

### 2.1. Participants

We analysed data from 52 participants (mean age *M* = 22.5, SD = 4.6; 23 females, 1 diverse), all with corrected-to-normal vision and with no reported neurological or psychiatric conditions. We included only participants who reported more than 10 years of exposure to Caucasian/European/White faces. The sample size was determined by a power analysis performed with the R package *pwr* [66]. This analysis returned a minimum of 48 participants required to detect a medium-sized effect of .25 for the relationship between face identity processing skills and typicality effects in a general linear model, with a power of .80 at the standard .05 alpha level. Therefore, we conservatively included 55 participants in initial testing. We then excluded data from three male participants from the analyses involving our main task (face detection task; FDT) and their relation to test battery scores: one was excluded due to lack of compliance with task instructions, another because FDT data were not saved, and a third due to substantially lower performance compared with the other participants’ mean performance (exceeding 3 SDs). We report and display the performance at the test batteries of 54 participants for the Jena-Bielefelder Famous Faces Test-2022 (J-BFFT-2022), of 53 participants for the Glasgow Face Matching Task 2 (GFMT2; only data from the non-compliant participant were excluded) and of 53 participants for the Yearbook Test (YBT) and the Cambridge Face Memory Test long version (CFMT+). In these cases, two different participants could not complete the tests, due to technical issues with the testing platform. Note, however, that their data were still included in the linear mixed models because we quantified their performance via mean rank on the remaining tests.

Before the experiment, all participants received information about the procedure and provided their written informed consent. The ethics committee (the name of the institution is hidden for peer-review, location: Germany) approved the experimental protocol (Reg. No. FSV 22/086) and the study was conducted in accordance with the guidelines of the Declaration of Helsinki. Participants received either monetary compensation or course credit for their participation. The study has been preregistered (https://osf.io/tc527/?view_only=c48d5a9d0b3447aca9bf58ff8b2db6d0). For transparency, we report a deviation in our study plan with respect to the preregistration. We decided not to perform a short familiarization task with some of the identities, in addition to the detection task, to restrict the focus of this study. The effect of familiarization with a subset of faces remains an interesting question for future studies.

### 2.2. Procedure

Participants performed an extended series of computerized tasks in a quiet room. Stimuli were shown on a BenQ BL2201 monitor (desktop resolution: 1680 × 1050; refresh rate: 59 954 Hz). All participants began with the FDT, since it was the most demanding in terms of reaction speed and attentional resources. The remaining tasks were performed in a randomized order for each participant and included the following tests: the long version of the Cambridge Face Memory Test [7], the Yearbook Test [67,68], a recently updated, computerized, short version of the Bielefelder Famous Faces Test (here called J-BFFT-2022), the Glasgow Face Matching Test 2—short version [69] and two tests assessing social cognition (The Tromsø Social Intelligence Scale [70] and the multiple-choice, computerized Movie for Assessment of Social Cognition [71]). The last two tasks are not included in the article. The overall duration of the testing session was about 2.5 h.

### 2.3. Experimental tasks

#### 2.3.1. Cambridge Face Memory Test long version (CFMT+)

The CFMT+ is a well-established test of face learning and memory ability, which can detect both cases of extreme deficit, as well as of excellent performance in face recognition [3,7,72]. We used the test version implemented in the online testing platform of the Applied Face Cognition Lab which has been used in several studies [5,10,64], as well as in applied contexts [5,73]. In the CFMT+, participants are first introduced to a set of black and white pictures portraying six unfamiliar young male faces and are instructed to memorize them. Then, triplets of faces are shown on the screen. In each triplet, only one face belongs to the previously learned ones, and the others are similar-looking distractors. The task is to indicate which of the three faces was memorized at the beginning of the test. The CFMT+ is composed of four blocks of increasing difficulty. While the task remains the same across its 102 trials, it increases in difficulty, as the stimuli display increasing variation in terms of viewing angle, image distortion, emotional expression, hairline display and repetition of distractor faces which thus also gain familiarity. The CFMT+ overall score consists of the number of correctly identified faces. The average duration of this test is 15 min.

#### 2.3.2. Yearbook Test (YBT—long version)

The YBT measures the ability to correctly match faces of the same persons despite age-related changes in their appearance [67]. The test includes eight full screen pages, each containing black and white pictures of different faces of the same gender. The top section of each page shows five young target identities, while the bottom section shows 10 old probe identities. We used a recently standardized, computer-based 40-items version of this test [68], containing images derived from the original test [67], of which only 35 items can be used (see Stacchi *et al*. [68]). Like the CFMT+, the YBT is implemented on the AFC Lab online platform. It is considered a highly challenging measure of face perception (identity matching) and it is used as the most diagnostic tool to identify Super-Recognizers [5,5]. All these probes are at least 25 years older than the young faces shown on top. Five of these probes are target identities, which are the same individuals as the young people shown on top, whereas the other five are foils. Participants are instructed to indicate (by choosing a number) which of the probe images corresponds to a given target face. The score represents the raw number of identities matched correctly throughout the whole task. This test takes about 20−25 min.

#### 2.3.3. Glasgow Face Matching Test 2—short version (GFMT2-S)

The GFMT2 measures simultaneous unfamiliar face matching ability [69], and represents an extended, more challenging, and recently validated version of the widely used GFMT [74]. Participants are presented with two faces, next to each other, on the screen, and must decide whether these belong to two different people or to the same person. The stimuli consist of colour pictures of unfamiliar faces drawn from the Glasgow Unfamiliar Faces Database. Compared with the original, the GFMT2 includes a more diverse set of ethnicities, viewing angles, distance-from-camera, image quality and emotional expressions, thus making it more realistic and difficult. We utilized two short versions of the GFMT2 (SA and SB), each including 40 items, administered in a counterbalanced order—each participant performed either version A or version B. The test lasts approximately 5 min.

#### 2.3.4. Jena-Bielefelder Famous Faces Test-2022 (J-BFFT-2022)

The J-BFFT-2022 is a test of familiar face recognition skills including celebrity faces, which is an adapted version of the BFFT [75,76]. We used a renewed, computerized, short version of this test, based on previous works [77–79]. Here, the J-BFFT-2022 (66 items) was split into two shorter versions of equal difficulty (33 items, J-BFFT-2022 short). Both test versions included face identities that were balanced in terms of face ethnic appearance and professional roles. Each short version lasts about 20−25 min (version randomized across participants). Each trial involved two phases: first, participants saw faces of celebrities on the screen, one at a time, and were asked to provide identifying information for each celebrity (e.g. name, other semantic information). If participants failed to recognize the celebrity from the face, they could leave the space blank. Independent of their response, each trial ended with the presentation of four name choices—the real name and three similar sounding foils. Participants had to either recognize the person’s name or select ‘I don’t know the person’.

Thus, this test allows us to distinguish correct identifications from the face from identifications from the name, reduces the risk of marking missing responses of unknown celebrities as miss, and also allows us to quantify general knowledge of celebrities/media exposure. The J-BFFT-2022 score, indicating face recognition ability, is computed as the number of celebrities identified via their face out of the total number of celebrities known by each participant. Note that the celebrities were selected through a pilot study run in 2021, thus making the test up to date to test the target population of this study (young German adults).

#### 2.3.5. Face detection task (FDT)

**Figure 1.**
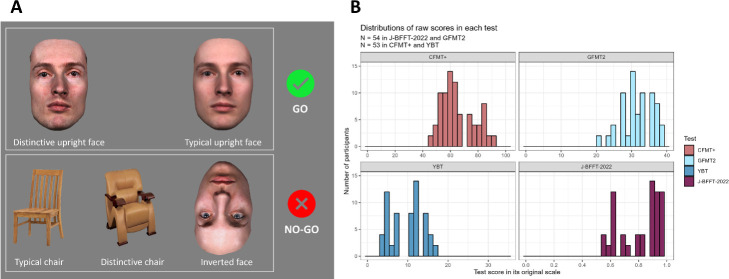
(*a*) Example of stimuli used in our face detection task. (*b*) Score distributions in each test of our face processing battery. The *y*-axis shows the frequency of scores (number of participants), whereas the *x*-axis shows raw scores (higher values reflect better performance). Warm colours indicate a higher task demand on memory and identification (CFMT+ and J-BFFT-2022), whereas cool colours indicate tests with higher demand of perceptual analysis of facial features in two simultaneously presented faces (YBT and GFMT2-S).

The FDT was designed to measure typicality effects on RTs to face stimuli, as well as their modulation over time. Participants saw images of upright faces, inverted faces and upright chairs, one at a time, at the centre of the computer screen, on a grey background. See figure 1*a* for examples of these images. Importantly, half of the target faces were typical and the other half distinctive. For a detailed description of the stimuli editing process and the typicality manipulation, see §2.3.1.1. Participants were instructed to provide speeded responses to upright faces only via a key on a computer keyboard. Each trial started with a white fixation cross at the centre of the screen (500 ms), followed by a target stimulus (which disappeared after response, and otherwise lasted maximum 1000 ms), a feedback screen (500 ms), which was shown in case participants responded incorrectly or too slowly, and another fixation cross (duration jittered between 195 and 1500 ms). Akin to a go/no-go task, participants were simply instructed to press a response key as fast as possible when they saw an upright face, and to refrain from responding whenever they saw any of the other two types of stimuli. The response key (either ‘f’ or ‘j’) was counterbalanced across participants. The FDT comprised 11 blocks, each with 48 trials, for a total of 576 trials. In total, participants saw 480 upright faces (go trials), 48 inverted faces (no-go trials) and 48 chairs (no-go trials), in a randomized order. Blocks were separated by a self-paced break, during which participants could monitor their cumulative performance up to that point (both RT and accuracy). Importantly, several aspects of this task were designed to avoid ceiling effects in RTs. First, we included a high proportion of go responses, which produced a response bias that participants had to inhibit, to keep accuracy high. Furthermore, when overall accuracy dropped below 85% participants received a warning message, encouraging them to be faster in the following block. Then, the presence of inverted faces made the task perceptually difficult. Finally, participants could see their performance in the past blocks, and were encouraged to try and beat their own records in the next block. These constraints were put into place to be able to detect a difference in RTs between typical and distinctive upright faces, which was our main effect of interest. Completing the FDT took approximately 15−20 min.

##### 2.3.5.1. Stimuli and typicality manipulation for the FDT

We used face stimuli drawn from the Jena three-dimensional face database (described in Müller-Bardorff *et al*. [80]), and utilized in previous studies (e.g. Itz *et al*. [78,81]; Limbach *et al*. [82]). Based on a previous typicality rating study, we selected 12 face identities (6 female and 6 male) of average typicality, to obtain faces with average typicality levels after morphing. This was done to ensure that later morphing would not result in extreme or unnatural effects. Each face had been photographed by four cameras simultaneously, and the resulting images were interpolated to generate a three-dimensional obj. file, including a mesh and a texture. To manipulate typicality, we generated caricatures and anti-caricatures (both in shape and texture), by morphing each individual veridical three-dimensional file with respect to a gender-matched average, using the morphing tool in DI3Dview™. Both the male and the female average faces were created using faces of the Jena three-dimensional database, using 44 and 49 face identities for the male and the female average, respectively. We subjected each face to both caricaturing (+0.33 units on the face trajectory, opposite to the face norm, where the norm is represented as 0.00 and the individual face as 1.00) and anti-caricaturing (−0.33 units on the face trajectory, in the direction of the face norm). The trajectory was defined for shape and texture separately: (i) shape trajectories were based on the pixel-wise distance, between each original face and the average face, of manually placed landmarks on the shape mesh of each face; (ii) texture distance was based on the differences, between each original face and the average face, in individual pixel’s colours. We created anti-/caricatures by shifting each face away or towards the average face along both trajectories. These shifts are expressed in percentage changes, which we took as references based on previous studies from our lab ([63,81,82]). The same shifts were applied to all faces. For each participant, we selected only one caricaturing version per identity (counterbalanced across participants), leading to six caricatured (distinctive) and six anti-caricatured (typical) identities for each participant. Since we were also interested in the effects of individual identities over time, we systematically tilted the individual faces according to a set of 10 camera angles (see electronic supplementary material, figure S1; see Itz *et al*. [81] and Ficco *et al*. [83] for details). Thus, each identity was shown from the same viewing angle four times throughout the task. This was done so that learning effects could be related to identity, as opposed to mere image processing (Jenkins *et al*. [84]). Finally, all face images were equalized in terms of luminance and spatial frequency using the SHINE_colour toolbox in MATLAB R2020a [85,86]. As specified above, 10% of the upright faces were shown inverted, which was simply achieved by modifying the frontal angled faces otherwise shown in the FDT. Chair stimuli were downloaded from the Internet and pre-processed to ensure similar image size, quality and a homogeneous background. We selected chairs high and low in perceived typicality based on a previous rating study, to include more variation and slightly increase task difficulty. Examples of the experimental stimuli are shown in figure 1*a*.

### 2.4. Data processing and analyses

First, we removed all RTs from the face detection task that were shorter than 200 ms [87], and we also removed RTs within individual participants which more than 2SDs higher or lower than the participant’s average RT. For each participant, we calculated indicators of face-space expansion and face-space adaptability during the FDT. We ran both full linear mixed models, in which the outcome variables are RTs or learning rates (for learning rates, see equations (2.1) and (2.2)). In our models, we included typicality (two levels: typical, distinctive), the aggregated face processing score or the test scores, and identity occurrence (continuous, ranging from 1 to the available identity presentations for each participant, max. 40) as fixed effects. Additionally, we modelled individual participants’ intercepts as random effects. Wherever model convergence allowed it, we also modelled random slopes of typicality within each participant (i.e. we allowed typicality to predict the outcome variable differently for each participant), in line with the idea that typicality perception reflects individual face-space organization. Our confirmatory analyses of individual differences include an aggregated face processing score (the participant’s mean rank on the scores of all tests), representing all face processing abilities. Our exploratory analyses of individual differences investigate the predictive power of each individual test separately. Note that the aggregated score is more robust to idiosyncrasies in any of the individual tests and is therefore taken as a main reference for our interpretation.

As for the face-space expansion indicator, we mainly estimated the two-way interaction between typicality and face processing when predicting RTs and learning rates with linear mixed models. Note that higher learning rates represent more efficient learning. We hypothesize that more expanded face-spaces would lead to increased differences in responses and learning efficiency between distinctive and typical faces, both favouring typical faces. Learning rates were calculated based on the following formula [88]; as cited in Leibowitz *et al*. [89], see equation (2.1)):


(2.1) $$RT_{n}=\;RT_{\infty }-\left(RT_{\infty }-\;RT_{0}\right)\cdot e^{-\alpha \cdot n}$$

from which learning rates to individual identities can be derived as follows:


(2.2) $$\alpha =-\;\frac{log\left(\frac{RT_{\infty }-\;RT_{n}}{RT_{\infty }-RT_{0}}\right)}{n}.$$

In equation (2.2), *α* represents the efficiency of learning, with larger values indicating more efficient learning, ${RT}_{\infty }$ is a constant term containing the minimum meaningful RT possible (when learning is approximating maximum; here set to 200 ms) [87], ${RT}_{0}$ is the RT the first^1^ time each identity is shown (when no learning has taken place yet) and ${RT}_{n}$ is the RT on the *n*^th^ presentation of that identity. We performed linear mixed models and tested the two-way interaction between typicality and the aggregated face processing score. RTs and learning rates are used as outcome measures for face-space expansion and adaptability, respectively. Note that, to account for potential non-linear trends in the time course of learning over time, in the case of face-space adaptability we test^2^ a model where the outcome variable is in log-scale and another with the outcome variable in its original scale.

All analyses were performed using R Version 4.2.0 and RStudio (R Core Team 2022), with the packages *lme4*, version 4.2.0 [91]*, lmerTest*, version 4.2.0 [92], *emmeans,* version 4.2.1 [93], *performance*, version 4.2.3 [94], *sjPlot,* version 4.2.3 [95], *bayestestR* [96] and *MQMF*, version 4.2.3 [97]. We fitted linear mixed models by restricted maximum likelihood estimation, and we obtained *p*-values for our effects of interest using multiple-comparisons corrected *t*-tests (Satterthwaite’s method). Also, to quantify the evidence of our effect of interest against the evidence of its absence, for each model we report (and interpret according to [98]) Bayes Factors (BF). Because we had no specific hypotheses about individual face processing tests, we conducted our analyses using the aggregated score (mean rank of the four face processing tests we used). We modelled gender as a covariate, since we detected a difference in RTs during the FDT between males and females in our sample (*W* = 990, *p* < .05). Females outperformed males in the CFMT+ (*W* = 1518, *p* < .05), the J-BFFT2022 (*W* = 1781, *p* < .005), but neither in the GFMT2 (*W* = 1317, *p* = 0.67), nor in the YBT (*W* = 1076, *p* = 0.25). Data and scripts related to this manuscript are available on OSF (https://osf.io/p5zqy/?view_only=e3fff060997e430e93f82c161eb64c73), and we report the formulas entered in our models in the electronic supplementary materials.

## 3. Results

We report a complete output of our analyses in electronic supplementary material, tables S1–S5, and on OSF. Here we only report the effects and interactions which reflect indicators of face-space expansion and adaptability, and their relation to face processing skills.

### 3.1. Test battery results

Test performance in our face processing battery is shown in figure 1*b* and descriptive statistics are displayed in table 1. Overall, our sample’s test results mostly compare with previously published data: [72, p. 3076] report similar CFMT + performance (*M =* 67.01, SD = 10.98) in 126 participants of similar demographics; [68, p. 8] report slightly lower performance in the YBT (*M* = 8.8, SD = 4.0, Median = 8) in an older sample of 252 participants, *M_ag_*_e_ = 29.0, SD = 12.7. Our sample performed better than that reported in [69, p. 257], who presented data from 108 participants (*M_age_* = 38.0, SD = 11.3). Finally, performance at the J-BFFT-2022 is similar to that reported in [78, p. 379], at a different BFFT version including 66 instead of 33 items (*M* = 0.87, SD = 0.10), and with a sample of 40 participants with demographics comparable with ours. Unsurprisingly, all our tests significantly correlate with each other, with correlations ranging between *r* = 0.19 (GFMT2 and J-BFFT-2022) and *r* = 0.43 (CFMT+and J-BFFT-2022), except for the GFMT2 and the J-BFFT-2022 (*p* = .056). See electronic supplementary material, figure S2, for the complete correlation matrix.

**Table 1.** Descriptive data of our test performance (cf. figure 2). SD = standard deviation.

test	mean	SD	median	range
*CFMT+*	66.31	11.15	65.00	47–90
*YBT*	9.59	3.24	10.00	4–17
*GFMT-S*	32.57	3.86	34	21–39
*J-BFFT-2022*	0.85	0.14	0.10	0.55–1.00

### 3.2. Overall typicality effects

On average, participants took 347 ms (SD = 31.6 ms) to respond, and their accuracy was high (0.9 (SD = 0.02)). Their hit rate was on average 0.99 (SD < 0.01) for the 90% Go Trials, but their mean false alarm rate for the 10% NoGo Trials was also high, 0.86 (SD = 0.09). Average sensitivity was high (*d*’ = 2.89) and all participants showed a bias towards responding even if an upright face was not present (*Criterion* = −2.65). Most importantly, given the lack of evidence for speed-accuracy trade-offs during the FDT (non-significant interaction between trial number and accuracy on RTs, *p* = .52), analyses were restricted to RTs as a measure of face-space expansion and adaptability.

Our hypothesis (iii), predicting shorter RTs during the FDT for typical, as compared with distinctive faces, was confirmed. On average, participants took 3 ms longer to respond to distinctive faces. The size of this difference is negligible (Cohen’s *d* = 0.09 [0.05–0.14]), but statistically significant (*t*(51) = 4.078, *p* < .001, note that 52 participants were retained for this analysis).

**Figure 2.**
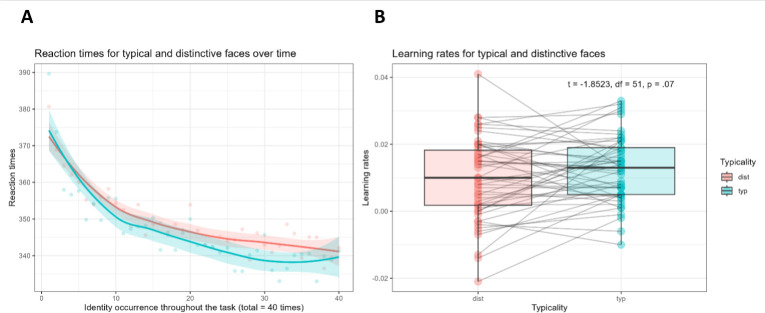
(*a*) RTs for typical and distinctive identities throughout the whole task (each identity is shown up to 40 times). (*b*) Learning rates for typical and distinctive identities throughout the whole task. Dots and connecting lines represent individual participants. Higher learning rates represent more efficient learning. The difference in learning efficiency between typical and distinctive faces is non-significant (*p* = .07).

Regarding hypothesis (iv), conversely, we found that typicality differences in RTs did not change over the course of the task, as reflected in the non-significant interaction between identity occurrence (i.e. identity repetitions) and typicality (*t*(23 910) = −1.529, *p* = .13). Visual inspection of the data (figure 2*a*) even seems to reveal an opposite trend, i.e. the difference of RTs between distinctive and typical faces slightly increases over time. As for our hypothesis (v), we did not find significant differences in learning rates between typical and distinctive faces, although there is a trend for higher learning rates for typical faces (*t*(51) = 1.852, *p* = .07, figure 2*b*).

### 3.3. Face-space expansion in relation to face processing ability

We did not find evidence for floor effects in RTs (potentially indicating ceiling performance) during the FDT. On average, participants took 348 ms (SD = 64 ms, Median = 340 ms, IQR = 83 ms) to respond to upright target faces.

**Figure 3.**
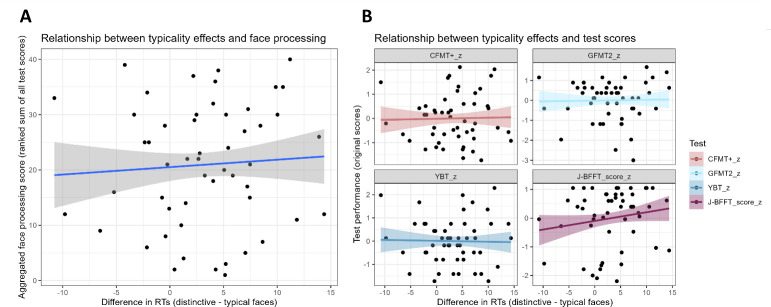
Non-significant relationships between the difference in RTs between distinctive and typical faces and face processing skills. (*a*) The *y*-axis shows the aggregated face processing score (mean of all test scores for each participant); (*b*) *y*-axis shows standardized performance in individual tests.

The linear mixed model including typicality and general face processing score as predictors of RTs reveal a non-significant interaction between the two factors (*t*(50) = −0.504, *p* = .617; BF = 0.02; electronic supplementary material, table S2). This suggests that the typicality difference, which is our index of face-space expansion, is not predicted by face processing skills (figure 3*a*), and that there is evidence in favour of the null model. When exploring this relationship by considering one test at a time, typicality and each individual test do not interact when predicting RTs, and the null model is supported in this case too (all *p*s > .201; BF = 0.01; electronic supplementary material, table S3).

### 3.4. Face-space adaptability in relation to face processing ability

**Figure 4.**
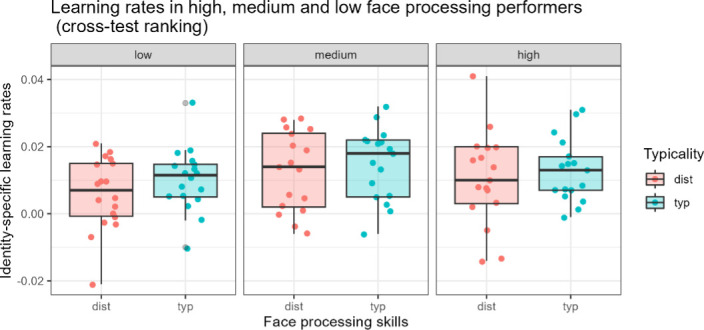
Difference in learning rates between distinctive and typical faces and face processing skills. The *y*-axis shows identity-specific learning rates, whereas the *x*-axis represents different performance groups (mean rank of performance in each participant); colour represents typicality. The difference between groups in learning rate, i.e. detection efficiency throughout the task, is not significant.

To test the combined effects of typicality and general face processing skills on face detection learning efficiency, we examined the two-way interactions between typicality and face processing using linear mixed models. The linear learning rate model (BIC = −624.9) fits the data significantly better than the log(learning rate) model (BIC = 241.9). In the best-fitting model, the two-way interaction of interest (typicality*face processing skills) is not significant (*t*(50) = 0.376, *p* = .708, *β* = 0.000). The BF approximated 0 (BF = 0.00), which suggests evidence in favour of the absence of the effect. In other words, high, medium and low performers in general face processing do not seem to differ as regards the detection efficiency between typical and distinctive faces (see figure 4; electronic supplementary material, table S4).

Considering each individual face processing test separately, none of the interactions involving this effect reaches significance (all *p*s > .06, all *β*s < 0.003; electronic supplementary material, table S5). The respective BF is very close to 0 (BF = 0.00), and suggests evidence for the absence of the effect. The interaction with the CFMT + only approached significance (*t*(44) = 1.926, *p* = .06, *β* = 0.003; pseudo-BF: 0.59). Descriptively, in this case the difference in detection efficiency between typical and distinctive faces is most pronounced in *medium* CFMT + performers with distinctive faces being less efficient than typical faces, as opposed to high and low performers (see electronic supplementary material, figure S3). However, our Bayesian analysis suggests that this effect is absent.

## 4. Discussion

### 4.1. Overview

In this study, we investigated the association between face identity processing ability and individual face-space organization, operationalized as detection differences between typical and distinctive faces. First, we replicated the detection advantage for typical over distinctive faces reported in prior studies. Second, face-space expansion, as defined here, was not predicted by face processing abilities. Third, face-space adaptability was not predicted by face processing abilities either. Overall, these results suggest that mental face representations (or, at least, the two face-space properties measured here) do not contribute to individual face processing skills, as opposed to what previous studies seem to indicate.

### 4.2. Typicality detection advantage and distinctiveness encoding advantage

Our result that typical faces are detected faster than distinctive ones dovetails with previous studies [40,56,57]. Such typicality advantage in detection appears to be smaller in size than that reported by [40,56,57]. This might be due to notable task differences between visual search in a complex visual scene [56], or in a face/non-face classification task [40,57,58]. Moreover, Dieciuc & Folstein [99] draw a theoretical difference between functional typicality, which is flexible and context-dependent, and structural typicality, which is the result of long-term exposure to facial appearance. Following this distinction, the difference in between typical and distinctive faces in this study might have been particularly small because participants’ typicality perceptions emerged throughout the course of a relatively short adaptation period—the task lasted about 20 min, therefore reflecting changes in functional, more so than structural typicality [99]. Nevertheless, all the above tasks including the present one required participants to perform fast decisions about the presence of an upright face. We thus interpret this typicality advantage as a general preparedness of the visual system to see faces that look closer to the average as more ‘face-like’ [100]. Note that our paradigm cannot distinguish between norm-based, and other models of face-space, such as the ideal representation model [52]. In fact, the processing advantage in favour of typical faces might occur both because they represent better ‘summaries’ of the category of faces (norm-based face-space [52]), and because they are more ideal representations of the category (ideal representation face-space) [52]. Future studies might adopt similar procedures to Trujillo and Anderson [52] to formally test these models against each other.

Contrary to our hypothesis, typical (as compared with distinctive) faces were descriptively associated with more efficient learning (*p* = .07). This might indicate that, with distinctive faces, there is less room and need for improvements in detection efficiency during the task. It is noteworthy that distinctive faces seemed to require less learning effort, although the task did not explicitly require identification, which might indeed take longer [86,101]. This suggests that, despite typical faces showing a detection advantage (in line with the idea that they are closer to the category prototype [102], distinctive faces might have a slight encoding advantage which then results in recognition advantages [63]. However, the difference is not statistically significant, so we conclude that there are no differences in learning efficiency between typical and distinctive faces in this task. Moreover, the power analysis for this study was not based on this effect, thus larger samples are necessary to further investigate this trend.

### 4.3. No relationship between face processing skills and face-space expansion

An important question was whether differences in RTs between distinctive and typical faces, which we interpret as indicators of face-space expansion, were related to face processing abilities. The current data clearly suggests that they are not, both when considering face processing skills in general and individual tests separately. This could be in potential contrast to the ERP data reported in Schroeger *et al*. [22], where typicality effects on the P200 were enhanced in high CFMT+performers. This apparent discrepancy between studies might be related to vastly different methods to quantify typicality effects, to different points in time at which these effects were assessed, or to a combination of these and other factors. For instance, the group differences in typicality effects on EEG signal reported in Schroeger *et al*. [22] lasted from 140 ms to about 260 ms after stimulus presentation, whereas our participants took on average 340 ms to detect a face. We also note that no differences between high and low performers were found in typicality ratings [22], supporting the idea that our behavioural indicators of face-space expansion might be not sufficiently sensitive. Moreover, Fysh [103] found no correlation between face detection RTs and accuracy in face memory and matching, suggesting that, more broadly, detection may require qualitatively different processing. However, in line with our conceptualization, face-space expansion might be a dynamic property more than a trait, which heavily depends on the degree of face variability one individual has been exposed to throughout their life up to the time of testing. To the degree that exposure to facial phenotypic diversity plays a major role in determining face-space expansion, as compared with personal face processing skills, it should not surprise that these have poor predictive power.

### 4.4. No relationship between face processing skills and face-space adaptability

Our second question was whether differences in learning rates between distinctive and typical faces, which we take as indicators of face-space adaptability throughout the task, were related to face processing abilities. Based on previous findings relating face memory performance to the size of adaptation aftereffects [47–49,104], we expected a positive relationship between learning rate-based face-space adaptability and test scores. On the contrary, the present study indicates that the two are not related, neither when considering general face processing skills, nor when considering individual face processing tests. This is at least partly determined by the very small learning rate typicality difference we were able to detect (see figure 2*b*). Interestingly, the only trend for a significant association between learning rates typicality differences and face processing emerged with the CFMT+, the same face learning and memory test used in two of the adaptation studies on which we based our hypothesis [47,48], both reporting significant associations. However, here, not only is this specific effect not statistically significant, its BF approximates zero and its beta estimate very low (*β* = 0.003), but it seems to be qualitatively different: in fact, if anything, learning differences seem to be larger in *medium* CFMT + performers, instead of high performers. However, the evidence is in favour of the absence of this effect, too. Notably, however, our sample (*n* = 55) is smaller than that of Dennett *et al*. ([47]; *n* = 78), that of Engfors *et al*. ([48]; *n* = 175) and that of Rhodes *et al*. ([104]; *n* = 240). Despite basing our sample size on a power analysis (whereas none of these studies report such calculations), it is possible that the power to find significant effects, at least with the CFMT+, was too low. However, the combination of the tests provides a more sensitive way of gauging individual differences in face processing, as opposed to the CFMT+ individually, such that two individuals ranking differently in four tests will be much more diverse than two participants with the same rank distance in the CFMT+ only. Thus, we still propose that, based on our data and analyses, differences in face-space adaptability do not explain differences in face processing abilities.

### 4.5. Limitations and open questions

We acknowledge some practical and interpretative limitations of the present study. First, at least in principle, our detection task could have been performed by just attending to a small portion of the screen (e.g. the eye region), which might have limited the typicality effects we observed. Second, we assume that all participants perceived our stimuli in a comparable way because of their exposure to similar faces, and that the typicality manipulation was large enough to produce effects. However, the visual difference between typical and distinctive faces may have been limited, at least in some participants, because we used smaller levels of caricaturing and anti-caricaturing compared with other studies ([63,82]). Note that we intended to create maximally realistic stimuli, and thus morphed faces with the average as in previous studies (for instance [22], and [54], although higher levels of caricaturing were also used, see [79]). Third, our morphing procedure produced increased blur in images of typical faces (cf. Figure 1). There are no theoretical reasons to expect that image blurring leads to detection differences. Moreover, any potential artifact would affect all participants equally, which would therefore not confound our analyses focused on individual differences. Nevertheless, future studies could employ a similar task to that described here with faces of pre-rated typicality, or to faces of the same- versus different other-‘races’ [56].

Finally, we note a few open questions. First, we acknowledge that the FDT is an indirect test of changes in face-space properties and thus may be insensitive to subtle changes in face representation. Future studies could compare participants’ typicality ratings for a large set of similarly generated stimuli, to explicitly test face-space expansion. These ratings could be collected before and after a period of adaptation to typicality levels that mirror those present among the stimuli, to test the extent to which these shifted following adaptation. Last, we intentionally did not prompt participants to the presence of caricaturing. In future studies, it would be of interest to determine whether the present effects at an individual level can be enhanced by a procedure which lets individual participants establish their own definition of distinctiveness in a separate task (e.g. see [105]).

## 5. Conclusions

We replicated detection advantages for typical faces, and found a trend for larger learning rates for typical (versus distinctive) faces in the context of a face detection task. Notably, the reported typicality differences in RTs were unrelated to independently measured face identity processing ability. While acknowledging behavioural measures’ relatively lower sensitivity for detecting variability in *face-space expansion*, our data does not suggest relation with individual differences in face identity processing. Similarly, concerning *face-space adaptability*, typicality differences in learning rates were also unrelated to face processing skills. We found a trend for most pronounced face-space recalibration in medium face memory performers (CFMT+), likely due to low power. Our findings suggest that neither face-space adaptability, nor face-space expansion, contribute to individual differences in face processing.

## Data Availability

The materials used to produce this manuscript are available on OSF [106]. Supplementary material is available online [107].

## Footnotes

^1^
In order to reduce data loss, in case the first time an identity is presented participants did not respond, we take the second time the same identity was presented instead.

^2^
The difference in the Akaike Information Criterion (AIC) and Bayesian Information Criterion (BIC) for models with versus without a quadratic term is > 2 [90]. We base our interpretations on the model without the quadratic term when describing the results of our face-space adaptability indicator if this fits the data better than the quadratic model. We report the BIC because it penalizes model complexity more.
